# Infant feeding, poverty and human development

**DOI:** 10.1186/1746-4358-2-14

**Published:** 2007-10-22

**Authors:** Annette Beasley, Lisa H Amir

**Affiliations:** 1 School of Social and Cultural Studies, Victoria University of Wellington, New Zealand.; 2 Mother & Child Health Research, La Trobe University, Melbourne, Australia.

## Abstract

The relationship between poverty and human development touches on a central aim of the International Breastfeeding Journal's editorial policy which is to support and protect the health and wellbeing of all infants through the promotion of breastfeeding. It is proposed that exclusive breastfeeding for 6 months, followed by continued breastfeeding to 12 months, could prevent 1,301,000 deaths or 13% of all child deaths under 5 years in a hypothetical year. Although there is a conventional wisdom that poverty 'protects' breastfeeding in developing countries, poverty actually threatens breastfeeding, both directly and indirectly. In the light of increasingly aggressive marketing behaviour of the infant formula manufacturers and the need to protect the breastfeeding rights of working women, urgent action is required to ensure the principles and aim of the International Code of Breastmilk Substitutes, and subsequent relevant resolutions of the World Health Assembly, are implemented. If global disparities in infant health and development are to be significantly reduced, gender inequities associated with reduced access to education and inadequate nutrition for girls need to be addressed. Improving women's physical and mental health will lead to better developmental outcomes for their children.

## Editorial

October 2007 has been dedicated by the Council of Science Editors to the global theme of 'Poverty and Human Development'. The Council's initiative follows earlier successes with simultaneous publications on a global theme across scientific journals. In 1996, 36 journals from 21 countries published articles on 'Emerging and re-emerging global microbial threats'. The following year, 97 journals in 31 countries published on the theme of 'Ageing'. The objective of this strategy is to raise awareness, and to stimulate research and international collaboration on important topics.

In a call for submissions on the current global theme of poverty and human development from the perspective of breastfeeding, the *International Breastfeeding Journal (IBJ) *referred potential contributors to the emerging policy development framework of the World Health Organization (WHO)'s Global Strategy on Infant and Young Child Feeding [1]. The relationship between poverty and human development touches on a central aim of *IBJ's *editorial policy which is to support and protect the health and wellbeing of all infants through the promotion of breastfeeding. In view of the significance of the global theme, the lack of response to the call for papers was disappointing. Nevertheless, it remains important that this journal endorse the intention of the global theme through editorial commentary.

The important issue of childhood survival has been highlighted in recent years, with statistics indicating 99% of childhood deaths occur in less-developed countries, and gaps between rich and poor within many countries are increasing [2]. *The Lancet *series on child survival reported that less than four in ten children are exclusively breastfed for 6 months (in the 42 countries accounting for 90% of deaths in under five year olds in 2000) [3]. There is high quality evidence that infants not exclusively breastfed are at increased risk of death from diarrhoea, pneumonia and neonatal sepsis [4]. It is proposed that exclusive breastfeeding for 6 months, followed by continued breastfeeding to 12 months, could prevent 1,301,000 deaths or 13% of all child deaths under 5 years in a hypothetical year [4]. In comparison, other individual preventive interventions, such as clean delivery, Hib vaccine and tetanus toxoid, could each prevent less than 5% of all child deaths [4]. Moreover, as Cattaneo and Quintero-Romero remind us: 'The deaths represent the tip of the iceberg: disease, malnutrition, suffering, admission to hospital, social and economic cost for families and communities add immensely to the death burden' [5] (p. 49).

**Figure 1:**
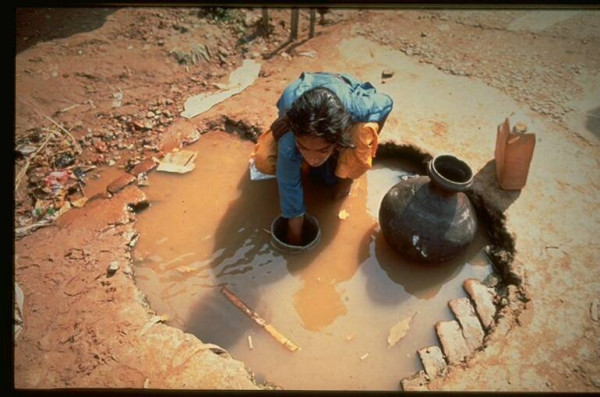
A woman collecting water in rural West Bengal, India.

**Figure 2:**
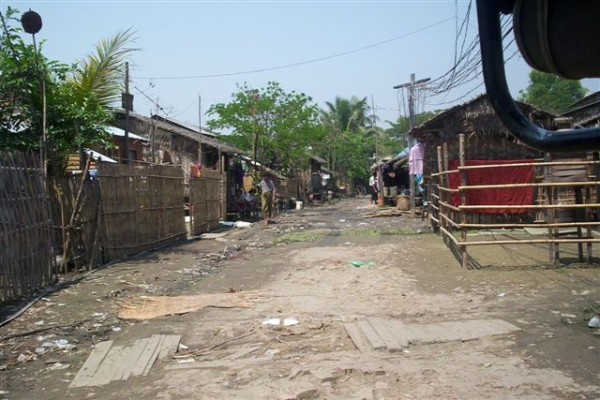
Peri-urban street in Myanmar (Burma).

**Figure 3:**
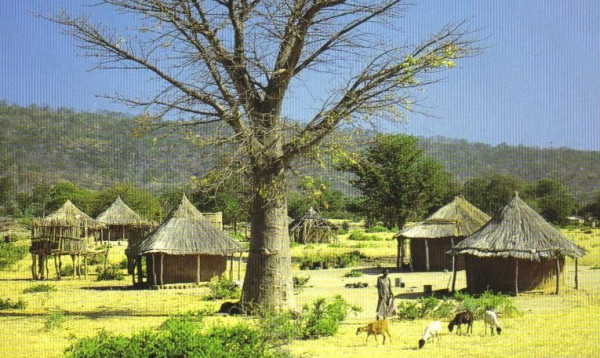
Village in Zimbabwe.

Recent natural events in Botswana draw attention to the risks associated with breastfeeding avoidance, a measure designed to eliminate the risk of postpartum transmission of HIV to exposed infants. Between November 2005 and February 2006, Botswana experienced unusually heavy rains and flooding. Normally considered a 'a middle income country, where infant formula is freely provided for a year [to all HIV-infected mothers] and the water supply has been generally regarded as safe' [6] (p. 2), the floods were accompanied by a huge increase in infant diarrhoea and mortality. In the first quarter of 2006, in just twelve health districts, there were 22,500 cases of diarrhoea, with 532 deaths in children under five (compared to 9,166 cases and 21 deaths for the entire country in the first quarter of 2005) [7]. The US Centers for Disease Control (CDC) found the public water supply was contaminated in 26 villages tested. The most significant risk factor for children with diarrhoea was not breastfeeding (adjusted Odds Ratio: 50, 95% CI: 4.5, 100). The risk factors for death included not being breastfed (OR 8.5, p = 0.04). Importantly, the HIV status of mothers and infants was not associated with the risk of death [7]. This finding, in common with other case studies reported at the 2006 WHO Technical Consultation on HIV and Infant Feeding [8] and at the Fourteenth Conference on Retroviruses and Opportunistic Infections held in Los Angeles this year [9-13], reinforces the need to be 'absolutely sure that replacement feeding is acceptable, feasible, affordable, sustainable and safe' [8] before suggesting that a woman with HIV - or indeed any woman - does not breastfeed her infant [6,14]. In many parts of the world, lack of access to clean water together with poor sanitation pose a serious threat to infant health and wellbeing; Figures 1, 2, 3 are illustrations of living conditions in Africa and Asia.

Among developed countries there is a conventional wisdom that poverty 'protects' breastfeeding in developing countries. Greiner was surprised when women in his 1975 study in St Vincent, West Indies, said they could not afford to exclusively breastfeed [15]. The researcher's explanation was that women had been influenced by the infant formula company brochures which instructed breastfeeding women to consume 3000 calories, illustrated with pictures of meat, fish and milk [15]. Since then, he has heard 'I could not afford it' from women in a range of countries (Greiner, personal communication, September 2007).

In her case-study of breastfeeding in Vietnam, Morrow argued that poverty actually threatens breastfeeding, both directly and indirectly [16]. For financial reasons, many women are required to return to work soon after childbirth; she found that even field workers were working by six weeks postpartum, leaving their infants to be supplemented with white rice porridge. Increasing male migration to the cities to seek employment, has led to further economic hardships for women, increasing their workloads physically and emotionally. Widespread use of breast milk substitutes - most commonly diluted sweetened condensed milk - and extensive advertising of infant formula compounded the risk of infant mortality and morbidity [16]. These concerns - poverty, gender issues, lack of breastfeeding and promotion of infant formula - resonate today.

Reflecting the debate on issues of social justice and human rights, the Global Strategy on Infant and Young Child Feeding identifies 'inappropriate feeding practices and their consequences' as 'major obstacles to sustainable socioeconomic development and poverty action' [1] (p. 2). The report warns that government efforts to 'accelerate economic development in any significant long-term sense' will not be achieved until 'optimal child growth and development, especially through appropriate feeding practices, are ensured' [1] (p. 2). However, as argued by the critics of income-focused indicators of poverty, effective promotion of optimal infant feeding practices among at risk groups require strategies that enhance individual capabilities and opportunities. Evidence of the potential effectiveness of such strategies is demonstrated by the findings of recent studies, which focus on the relationship between indicators of maternal deprivation, and infant care and wellbeing.

The determinants of growth failure among 12-24 month old children from an urban slum in East London, South Africa were examined using a case-control study [17]. The researchers compared 100 control children with 50 children with growth failure. The researchers found no significant differences between the two groups with respect to the incidence of disease and infection, indicators of household food security, health service utilization and environmental conditions [17]. However, growth failure was associated with the mother's caring capacity. Her caring capacity was largely determined by her background (those not born in the area had looser support networks), family status (children in the growth failure group were at higher risk of living in a household where the mother lacked decision-making powers), her educational status, her nutritional knowledge and her health (general and reproductive) [17].

The effect of maternal depression on infant health in a low-income country was investigated by Rahman and colleagues [18]. They followed a cohort of 632 physically healthy women in a rural community in Pakistan. Maternal depression in the prenatal and postnatal periods predicted poorer growth and higher risk of diarrhoea in these infants [18]. The association remained significant after adjustment for birth weight, economic status, maternal age and literacy, infant's sex, and family structure [18]. Other studies in Goa and Tamil Nadu in India confirm these findings [19].

In Nicaragua, the link between poverty and infant survival was explored through examination of the reproductive histories of a representative sample of 10,867 women [20]. This study confirmed the relationship between maternal deprivation and infant mortality through identifying a 'pattern of very high infant mortality in a situation of low maternal education and social inequity' [20] (p. 66). In particular, the study found 'the highest infant mortality risks were . . . in poor households in a non poor environment rather than in poor households in a predominantly poor environment' [20] (p. 65). This finding led the authors to conclude that 'inequity in the satisfaction of the household's basic needs seemed to increase the mortality risks of the infants' [20] (p. 66).

Michael Marmot has also emphasised the importance of maternal education and social factors in reducing infant death [21]. The state of Kerala is one of the poorest states of India viewed according to the poverty indicator of income. Yet, the state infant mortality rate was 17 per 1,000 live births in 1990 - much lower than the infant mortality of 65 per 1,000 in the top 25% richest income states [21]. Kerala has medical facilities in the majority of villages, but also much higher female literacy (65% women were literate in 1999 compared to 11 to 36% in five other Indian states). Marmot suggests that while education itself is important, it also indicates that women are highly valued. 'A society that invests socially in women as well as men is one that is more socially inclusive' [21] (p. 187). This leads to the idea that social capital, or social cohesion, leads to better health [22].

Anderson and colleagues examined the relationship between social capital (defined here as the existence of community networks, civic engagement, local identity and a sense of solidarity and equity with other community members, and trust and reciprocal help and support) and breastfeeding in low-income Puerto Rican women living in the United States [23]. They concluded that 'women with more social capital were more likely (Odds Ratio 2.25, 95% CI: 1.02, 4.95) to have breastfed the index child, suggesting that social capital is an important predictor of breastfeeding initiation in this community' [23] (p. 39). Further research is needed to establish whether the concept of social capital is useful when assessing determinants of breastfeeding in other communities.

Poverty is a gender issue. 'Women constitute 70% of the world's 1.3 billion absolute poor. They earn only 10% of the world's income, own one percent of the world's property, but they work two-thirds of the world's working hours' [24] (p. 3). Early marriage and consequent early childbearing, accompanied by lack of adequate nutrition has adverse effects on women's health. Exclusive breastfeeding is particularly difficult when women have the double burden of paid work as well as being expected to do the majority of the domestic work. The work women do in feeding their children by producing breast milk is not recognised, although it has great economic benefit for the family and the wider community [24]. Reproductive rights are needed to enable couples access to contraception (where lactational amenorrhea is no longer appropriate) as longer birth intervals can play an important part in improving infant, child, and maternal survival [25].

Gender issues such as sex discrimination occur even before birth. Sex-selective abortion is well documented, particularly in India and in China, where 17% more boys are born than girls, resulting in an estimated excess of 1.7 million male babies per year [26]. An estimated 60 to 100 million girls are 'missing' worldwide [27]. Childhood mortality is also higher for girls among cultures where there is a strong preference for sons. In India, girls have a 40% greater risk of dying between the age of one and five years than boys [2]. Inferior childhood nutrition can lead to a lifetime of health problems; the risk is high for women and girls in cultures with a strong preference for sons.

The findings of the studies cited above are not unique. Less than optimal infant growth, and the higher risk of infant morbidity and mortality among those raised under conditions of poverty and deprivation is well established. Not so widely acknowledged, however, is that such infants are denied basic human rights. That is, they are deprived not only of the right to an adequate standard of living, adequate food, clothing, and housing but also the more fundamental human right of freedom from hunger [28]. This issue is taken up by Kent, who argues that 'The human rights of children with regard to their nutrition must be located within the broader context of the human right to adequate food in modern international human rights law and principles' [29] (p. 2). Kent contends that in situations where infants are at risk from less than optimal growth associated with poor food security and inadequate sanitation, their rights take on a further dimension [29]. That is, the right to optimal nutrition actioned through acknowledgement of the right to be breastfed.

Over the past few decades the right to be breastfeed among the world's poorest populations has been undermined by the well publicised tactics of the infant formula industry [30-32]. Concerned over the alarming increase in rates of infant malnutrition and mortality associated with the promotion of artificial feeding as 'modern', 'scientific' and 'progressive' among the poor of the developing nations, the WHO developed the International Code on the Marketing of Breast milk Substitutes in 1981 [33]. In many countries compliance with the code remains voluntary rather than regulated, and vulnerable infants continue to be placed at risk.

Central to the ethics surrounding the marketing of infant formula is the right of the infant to be breastfed 'in the sense that no one may interfere with their mother's right to breastfeed them' [29]. Earlier this year, *IBJ *outlined its editorial policy that manuscripts sponsored by infant formula manufacturers would not be accepted [34. ]A key ethical concern associated with industry funding, sponsorship and support of breastfeeding research is the potential for biased results to be utilised in marketing strategies that target vulnerable populations. Among responses received endorsing the new policy was an email from a breastfeeding advocate in Manila, Philippines. Attached to the message were images of advertisements from several Manila daily newspapers advertising 'fortified milk' as a protection from diarrhoea and pneumonia among infants aged between 12 and 36 months. The correspondent was particularly concerned about the simultaneous publication in the Manila press of the trial results and the advertising campaign, both of which featured the lead researcher. Our correspondent reacted to the advertising campaign with the question, 'Who can stop this kind of industry manipulated efforts?' In view of the constraints of a global market economy, a definitive response to such a question is almost impossible. However, it is clear that such practices breach the principles of justice, equity and human rights.

Countries such as the Philippines have faced declining breastfeeding rates and increasingly aggressive marketing of infant formula over recent years. At present, the Health Department in the Philippines is trying to restrict the widespread advertisements which proclaim that infant formula 'makes geniuses out of babies, promotes love and affection, promotes family' [35]. The response from the Pharmaceutical and Healthcare Association of the Philippines was to sue the Health Department on the grounds that only congress has the authority to change regulations. The situation has been compounded by the intervention of the chief of the U.S. Chamber of Commerce who wrote to Philippine President Gloria Macapagal Arroyo urging her to re-examine the Health Department's plan or risk the country's 'reputation as a stable and viable destination for investment' [35]. In June of this year, the Supreme Court ordered a halt to the implementation of stronger advertising regulations [35]. The implications of these events and the potential for repercussions across other developing countries in Asia are worrying.

The Philippines example illustrates that it is essential that governments around the world adopt the recommendations from the *Innocenti Declaration *(adopted in 1990 and reaffirmed in 2005) [36]. In the light of increasingly aggressive marketing behaviour of the infant formula manufacturers and the need to protect the breastfeeding rights of working women, urgent action is required to ensure the principles and aim of the International Code, and subsequent relevant resolutions of the World Health Assembly, are implemented [5,33]. As individuals, women are powerless to counter the complexity of societal forces that interfere with exclusively breastfeeding their infants for six months. What is required are 'structural changes . . . to society that will enable all mothers to breastfeed with assurance and safety' [24] (p. 17), including full implementation of the ILO Maternity Protection Convention [5,37]. If global disparities in infant health and development are to be significantly reduced, gender inequities associated with reduced access to education and inadequate nutrition for girls need to be addressed. Improving women's physical and mental health will lead to better outcomes for their children. More importantly, 'Inequalities in health between and within countries *are *avoidable . . . Reducing these social inequalities in health, and thus meeting human needs, is an issue of social justice' [38] (p. 1103). Until such time that established poverty indicators are addressed and eliminated, the opportunity of optimal growth and development among at risk infants will continue to be denied.

## Competing interests

The author(s) declare that they have no competing interests.

## Authors' contributions

AB wrote the first draft of the paper. LHA contributed to subsequent drafts.
